# Collective Knowledge and the Limits of the Expanded Identification Doctrine

**DOI:** 10.1093/ojls/gqae025

**Published:** 2024-07-12

**Authors:** Alexander Sarch

**Keywords:** Corporate crime, knowledge, mens rea, collective knowledge doctrine, encouraging, group belief, informational abuse, Economic Crime and Corporate Transparency Act 2023

## Abstract

The Economic Crime and Corporate Transparency Act 2023 expanded the identification doctrine in welcome ways, but, I argue, does not go far enough. Specifically, I contend that the Act’s reforms do not sufficiently respond to the threat of senior managers who culpably interfere in the proper flow of information within the company to orchestrate harmful or risky practices by others, all while seeking to avoid liability by preventing any individual from forming the full *mens rea* of any economic crime. How should the criminal law respond to this gap? I argue it would be problematic to respond by extending individual liability even further—say, by expanding the already ‘disturbingly wide’ inchoate offences in the Serious Crime Act 2007. Instead, the collective knowledge doctrine provides a tailor-made solution to these scenarios. This doctrine would permit courts (in narrow circumstances) to aggregate individuals’ mental states within the company to construct a distinct corporate *mens rea*. I argue that section 196 of the 2023 Act, which expands the identification doctrine, could be read to incorporate a narrow version of the collective knowledge doctrine—at least if courts are willing to adopt a purposivist orientation aimed at giving effect to the wider aims of Parliament. A restricted version of the collective knowledge doctrine would have normative benefits and so, I suggest, is worth putting to the courts through test litigation.

## 1. Introduction

The identification doctrine has recently received a major upgrade. It is the chief mechanism in English law for attributing crimes to organisations and a key tool for combating harmful criminal behaviours in corporate settings. Long criticised as too narrow,^1^ the doctrine has finally been expanded via the Economic Crime and Corporate Transparency Act 2023 (the 2023 Act).^2^ Among the 2023 Act’s changes to corporate criminal liability, two of the most central are: (i) adding a new corporate offence of failure to prevent fraud, and (ii) expanding the identification doctrine to cover senior managers who commit economic crimes.^3^ These improvements are due in no small part to the proposals offered in the Law Commission’s 2022 Options Paper.^4^

These reforms have been broadly welcomed,^5^ and rightly so. Nonetheless, this article argues they do not go far enough in important respects. The difficulty is that both the new failure to prevent offence and expanded identification doctrine remain committed to an individualistic framing. That is, they embody the premise—labelled the Individualist Constraint below—that a single natural person within the company must be identified who committed the offence and possessed the requisite *mens rea* in order for the company to be inculpated.^6^ However, this premise faces normative and conceptual problems. There are important differences between the mental states of natural persons, which are real psychological states, and the mental states the law attributes to an artificial entity like a company, which are legal constructions. The 2023 Act, to the extent it remains committed to this Individualist Constraint, does not go far enough. I argue that the Act’s reforms do not sufficiently respond to the threat of senior managers culpably interfering in the proper flow of information within the company as a way to *orchestrate or enable* harmful behaviours by others that may benefit the company while avoiding the prospect of liability by preventing any individual from forming the full *mens rea* for any economic crime.

The possibility of these worrisome cases was recognised by the Law Commission, which put the point (albeit cautiously) thus:

We accept that devolved structures are not necessarily put in place *in order* to avoid corporate responsibility, criminal or otherwise, but given that such devolved structures *are* put in place by large corporations, it is reasonable to ask how the decisions and conduct that flow from such decisions of the corporation should be treated by the criminal law.^7^

In describing the worry about corporate structures that function to help avoid corporate liability as ‘not necessarily’ intentional, the Law Commission leaves open that such structures could sometimes be intentional efforts to avoid liability—or at least be recklessly (or negligently) allowed by management to take root. This article takes seriously the worry that such culpably created structures have the effect, whether intentionally sought or recklessly allowed, of enabling culpable companies to avoid liability under existing doctrine. The underlying trouble is that deliberate informational interference by management can facilitate risky or harmful behaviours lower down in the organisation, but corporate liability is effectively blocked because no individual crime is committed or assisted thanks to the lack of any individual who possesses the whole *mens rea* for such crimes. I argue that the 2023 Act, to the extent it remains committed to a strictly individualist approach (requiring an individual with full *mens rea*), does not adequately respond to the threat of management engaging in this kind of informational interference as an intentional or reckless way to help the company avoid liability.

Nonetheless, there is a doctrine familiar to corporate criminal law practitioners and scholars that provides a tailor-made solution to these cases: the collective knowledge doctrine.^8^ As will become clear, it permits courts to aggregate the mental states of individual employees within the company to construct a distinct corporate *mens rea* as needed for a corporate conviction. This doctrine has been controversial,^9^ but largely, I suggest, because it has been considered mainly in an unrestricted form that permits aggregation without limit. That would indeed be objectionable. Instead, such a potent doctrinal tool should be deployed only within strict limits where there is a particular need for it: namely, only in response to highly culpable forms of informational abuse by the organisation and only as a gap-filler when individual liability does not suffice. Within appropriate limits, the collective knowledge doctrine is normatively desirable and defensible. At least, this is what I have argued previously with respect to US federal corporate criminal law^10^ and continue to maintain here. This article suggests that the collective knowledge doctrine is ripe for a fresh look given the under-inclusiveness that remains even after the 2023 Act’s expansion of corporate criminal liability.

The first and most important aim of this article is to make the theoretical case for the under-inclusiveness of the 2023 Act with respect to informational abuses in organisations.^11^ After sections 2 and 3 provide necessary background, section 4 argues that the 2023 Act does not go far enough in addressing cases of culpable informational interference. Even with the broad scope of individual inchoate liability under sections 44–6 of the Serious Crime Act 2007, violations of which can now be attributed to the company if committed by a senior manager, certain highly culpable forms of interference by management are still likely to escape liability—meaning that the company will also avoid conviction even under the expanded identification doctrine. Specifically, I argue that neither the Act’s new failure to prevent offence nor the expanded identification doctrine suffices for convicting the company in problem cases where senior management culpably interferes to compartmentalise knowledge with an eye to benefiting from harmful practices while avoiding liability by preventing any individual from forming the *mens rea* of an economic crime.

Accordingly, the aim of the second half of the article is to consider how the criminal law should respond to the liability gap that remains following the 2023 Act’s reforms. One response, considered in section 5, is to double down on the individualist approach and expand the scope of the individual offences that senior managers engaged in culpable informational interference could personally be convicted of—chiefly, the inchoate offences in the Serious Crime Act 2007. However, I argue there are serious drawbacks to this approach. Besides requiring another statutory amendment, I argue that expanding individual liability even further beyond what is covered by the existing, ‘disturbingly wide’^12^ section 44–6 inchoate offences is the wrong way to go. It would improperly sweep up individuals in corporate settings into the net of criminal liability even though they have little personal culpability.

An alternative response, explored in section 6 of the article, is to abandon the Individualist Constraint and take seriously the task of legally constructing a distinct corporate *mens rea* as a narrowly tailored response to culpable interference in the proper flow of information within the organisation. That would be to adopt a restricted form of the collective knowledge doctrine that permits convicting the organisation itself but only in response to a culpable organisational structure where the individual bits of misconduct do not rise to the level of criminality in isolation. This is a normatively attractive solution to the cases where the expanded identification doctrine falls short.

Whether the text of section 196 of the 2023 Act, expanding the identification doctrine, permits the judicial adoption of this solution, however, is a more complicated matter. As discussed below in section 6, there are textual hurdles, but I suggest that the Act nonetheless can be interpreted to allow a restricted version of the collective knowledge doctrine. Such a reading would provide a normatively attractive solution to the problem cases from section 4 while also not requiring new statutory reforms. Still, it would require a form of purposive interpretation that may not find favour in all camps. Accordingly, when it comes to the viability of the collective knowledge doctrine as a litigation strategy, I offer only a conditional conclusion: if one thinks the courts are sufficiently purposivist in orientation (a matter I cannot resolve here), then there would be a weighty case for the judicial adoption of a suitably restricted collective knowledge doctrine. Mapping out what this case looks like is the aim of section 6. As the 2023 Act is hot off the press and the case law interpreting it not yet solidified, this is an issue worth putting to the courts through test litigation. The collective knowledge doctrine is worth keeping in the prosecutor’s toolbox as courts grapple with the limits of this new legislation.

## 2. The Collective Knowledge Doctrine: Background and Necessary Limits

To provide background for the ensuing discussion, this section reprises the normative case for the collective knowledge doctrine by drawing on arguments I have previously offered regarding US law. Looking to US law makes sense here since American courts and commentators have given some of the most sustained consideration of this doctrine. To see the puzzle it responds to, consider the following hypothetical,^13^ of which this article will consider numerous variations.^14^

Separate Knowledge (Basic): Big Oil Corp is charged with knowingly making false statements to a branch of the government (a crime in most jurisdictions, eg 18 USC § 1001 in the United States or section 2 of the Fraud Act 2006 in England & Wales). Two teams within Big Oil Corp, A and B, collaborate to submit a report to a regulator asserting that P, which let us suppose is a statement about the emissions levels of a plant operated by the company. It turns out that P is false. No employee in Team A or B knows (or believes) that not-P. One employee in Team A, Alice, knows that P entails Q, but has no knowledge or belief about whether Q is true. Another employee in Team B, Betty, knows that not-Q, but does not know that P entails Q. Betty and Alice never talk or combine their knowledge, so neither forms the belief that not-P.

The puzzle is: should Big Oil Corp be convicted of violating the relevant statute, which would require some form of knowledge of the falsity (or at least the risk of falsity) of the statement in the report that not-P?

Most US courts that have considered the issue answer ‘no’ (and not only in criminal contexts, but also in civil contexts).^15^ Instead, most—though arguably not all^16^—US courts adopt an individualist approach when applying the *respondeat superior* rule that represents the dominant US approach to attributing liability to corporations. Under *respondeat superior*, when an employee commits a violation, the violation (including its mental state) can be imputed to the employer company if, at the time of the violation, the employee (i) intended to benefit the company in so doing and (ii) was working within the scope of her employment.^17^ When applying this rule, most US courts reject the notion of collective knowledge and instead adopt the following individualist premise:

**Individualist Constraint**: An organisation possesses knowledge that p as required for being guilty of a given crime only if there is an individual employee or member of the organisation who, acting within the scope of her employment and for the organisation’s benefit, legally counts as knowing that p (ie either actually believes p is a practical certainty or was wilfully ignorant as to p).^18^

I have argued against this premise in prior work.^19^ While the requirement has sensible motivations, including concerns about vagueness and fair notice, it does not address the threat of corporations that *culpably structure their operations to prevent the sharing of information*, where this makes it less likely that any individual will form the *mens rea* for crimes (like fraud), but still enables the company to benefit from risky or harmful conduct.^20^ Instead, I have argued that a narrow collective knowledge doctrine is an attractive solution to this problem.^21^ The core proposal is to adopt a rule like the following:

**Restricted Collective Knowledge Doctrine**: Imputing a piece of knowledge, K, to the organisation—even though K was not possessed by and could not be imputed to any individual within the organisation—is justified if (i) its employees (acting for the organisation’s benefit and within the scope of their employment) culpably interfered with the proper flow of information (which otherwise could have led other appropriate employees to acquire K) and (ii) this interference was sufficiently culpable to render the organisation as culpable as it would have been had an individual possessed K and the organisation acted analogously.

There are two important caveats about the proposal. First, I do not endorse knowledge aggregation for cases of non-culpable ignorance. This can be seen in the following variation of the example above:

Separate Knowledge (Innocent): Assume the same set-up as Separate Knowledge (Basic) applies, but the reason neither Alice nor Betty (nor anyone else in their teams) conclude that not-P is not the fault of anyone in the company. Rather, the two teams were responsible for different aspects of the company’s operations and fed into different parts of the report. They were not expected to review or collaborate on each other’s sections or combine their knowledge. The teams did not normally work together, and so Alice and Betty would not have reasonably been expected to share information. The fact that the falsehood was not picked up was not the fault of any individual (even if a careful reviewer with both Alice’s and Betty’s knowledge could have identified the falsehood).^22^

Knowledge aggregation would not be appropriate here. Instead, aggregation should be an exceptional legal response to culpably induced ignorance within the company and used as a basis for imputing *mens rea* to, and thus allowing conviction of, the company only (not an individual).

Second, it is important to note that knowledge aggregation is not *needed* in cases of individual wilful ignorance. Where a manager is genuinely wilfully ignorant of an inculpatory fact, existing doctrine allows knowledge of it to be imputed to the manager, which in turn enables convicting the company through traditional routes (*respondeat superior* in the United States or the identification doctrine in English law).^23^

Accordingly, the collective knowledge doctrine actually is necessary and justified only in cases of sufficiently culpable interference with information flow in the company where this does *not* amount to wilful ignorance of relevant facts by any individual. An example of the sort of problematic structure that demonstrates the need for the collective knowledge doctrine (which I will argue the 2023 Act does not adequately address) is this:

Separate Knowledge (Culpable Interference): Assume the same set-up as Separate Knowledge (Basic) applies, except now Alice and Betty’s teams *do* routinely collaborate on projects like this. Normally, the two teams would talk and share their knowledge, but management interferes with the proper flow of information in the company. Management knows members of these teams, including Alice and Betty, tend to poke around looking for legal problems, and this has cost the corporation significant amounts of money in the past. So, management—while generally having nothing to do with submitting such reports and having no specific suspicions about anything in this report—tries to separate Alice and Betty’s teams by: (i) placing their offices in different buildings; (ii) giving Team B another major assignment so they have less bandwidth to scrutinise Team A’s work; and (iii) sending the teams confusing instructions on how to access existing datasets they might wish to examine for this report, etc. Management hopes this will prevent the two teams’ members from talking and discovering problems with the company’s business practices. Thus, Alice and Betty never talk about the report, and they never draw the inference that not-P. They are both victims of management’s scheme to compartmentalise potentially damaging information. Management creates a structure designed to prevent people like Alice and Betty from sharing information, in the hopes of preventing knowledge of legal violations or harms in general from being noticed so the company can have plausible deniability. Thanks to management’s culpable interference, neither Alice nor Betty (nor any other member of their teams) actually comes to believe or suspect that not-P.

Crucially, no one in this example was wilfully ignorant under US law.^24^ After all, management lacked suspicions about the particular risk that the submitted report contained falsehoods. They created generalised informational barriers, but lacked *particular suspicions* about the report (or similar violations), as needed to be wilful ignorant of the fact that the report contained falsehoods.^25^ Here, there is a need for knowledge aggregation to respond to the culpable interference of management in the proper information-sharing procedures of the company. I have argued elsewhere that, much as the equal culpability of wilful ignorance and knowledge can support imputing knowledge to an individual who technically lacks it, there is likewise a plausible *equal-culpability basis* for aggregating Alice and Betty’s mental states in order to impute knowledge that not-P to the company (even if not to any individual).^26^ The idea is that compartmentalisation or other informational interference by management (even when not amounting to an individual wilfully preserving their own ignorance) can entail the same degree and type of culpability *for the organisation* as if an individual employee had known that not-P, and this culpability provides a basis for imputing such knowledge *to the organisation* (even if not any individual).

Having outlined why the collective knowledge doctrine is needed against the backdrop of US law, I turn now to my primary question: would English corporate criminal law, especially with the recent reforms, fare better with respect to these cases? I will argue the answer is ‘no’. Cases like Separate Knowledge (Culpable Interference) remain a problem even under the 2023 Act.

## 3. The Old Identification Doctrine

Prior to the 2023 Act, it is obvious that the law would not have allowed a conviction of the organisation itself in cases like the above. This is because English corporate criminal law traditionally has been highly individualist in orientation.^27^ To appreciate the ambition of the 2023 Act’s reforms to corporate criminal lability, recall the law as it stood previously.

The primary mechanism for imposing direct criminal liability on an organisation was—and remains—the identification doctrine. The 2023 Act widened it. Under the identification doctrine, a company can be convicted of a crime only if an officer or manager of the company who is sufficiently senior to actually *be* the company—its directing mind and will (DMW)—possessed the *mens rea* of the offence. If this actor does the *actus reus* or causes its commission while possessing the requisite *mens rea*, the company will be deemed to have committed the offence.

The question is who counts as a DMW. *Tesco Supermarkets Ltd v Nattrass* characterised a DMW as someone who has full discretion and authority to ‘control what [the company] does’.^28^ From *R v Andrews Wetherfoil Ltd*,^29^it was clear that not every high executive acting for the company was a DMW. On Lord Reid’s explanation in *Tesco*, ‘[n]ormally the Board of Directors, the Managing Director and perhaps other superior officers of a company carry out the functions of management and speak and act as the company. Their subordinates do not.’^30^ Similarly, Viscount Dilhorne explained the key is ‘to determine … who [is] … in actual control of the operations of the company’.^31^ The identification doctrine was an exceedingly narrow test. *SFO v Barclays*^32^ clarified that even the CEO and board members might not constitute a DMW with respect to particular transaction if their authority to undertake it was not absolute (eg if it must be ratified by the full board).

Because it will be important later (section 6), note there is some uncertainty about whether the identification doctrine requires the same DMW to both carry out the *actus reus* and possess the *mens rea* in order to convict the company of the offence.^33^ The narrowest version of the doctrine would allow convicting the company only if the whole offence is committed (both *actus reus* with *mens rea*) by a single DMW. A broader version would allow the *actus reus* to be done by someone else within the company than the DMW who possessed the *mens rea*—particularly if the DMW with *mens rea* directed or ordered the *actus reus* to be committed by another (who may have lacked *mens rea*), or was aware of the commission of the *actus reus* and did not intervene to block it. As argued in section 6A, some cases suggest the broader version is correct.^34^

The old identification doctrine faced extensive criticism. The most serious problem was its narrowness: the doctrine made it too difficult to convict corporations that warranted it.^35^ Only the mental states and conduct of someone with *ultimate authority* (not just some authority) inculpated the corporation. As with *respondeat superior*, the identification doctrine was committed to the Individualist Constraint: a single individual must be found who had the requisite *mens rea* of the crime for the organisation to be guilty of the offence. However, the old identification doctrine was even more problematic because it also took attributing offences to an organisation to be possible only for the acts and mental states of an extremely narrow set of individuals: a DMW. Accordingly, the doctrine did not reflect the realities of decision making within modern large companies, where for ‘perfectly sensible organisational reasons the Board and senior management are … not in possession of the granular information required … to participate in, let alone acquire *mens rea* with respect to, individual, allegedly criminal transactions’.^36^ It is this failure to reckon with the complexity of how information is shared and decisions taken within large companies that gives rise to the cases of culpable informational interference that this article focuses on. I argue that they remain a problem even after the 2023 Act’s reforms.

## 4. The Recent Reforms Don’t Eliminate the Problem of Culpable Interference in Information Flow

This section presents my primary argument that the reforms in the Economic Crime and Corporate Transparency Act 2023 do not go far enough when it comes to informational abuses in organisations. The Act’s key corporate liability provisions were animated by the aim of ‘holding corporations liable in their own right for economic crime’ and discouraging them from turning a blind eye to and profiting from such crime.^37^ The 2023 Act contained two major changes to corporate criminal law in the UK^38^ that one might think help address the problem cases from section 2 to which the collective knowledge doctrine speaks: (i) a new failure to prevent fraud offence (to be brought into effect by statutory instrument^39^); and (ii) a substantially expanded identification doctrine for economic crimes (to take effect two months after the passage of the Act on 26 October 2023^40^). This section argues that neither reform suffices to fully address all versions of cases like Separate Knowledge (Culpable Interference). This is because of the unique nature of the wrong in question: carefully designed conduct that facilitates harmful corporate practices but enables liability to be avoided by blocking any individual from forming the *mens rea* of a particular crime.

### A. Solution 1: New Failure to Prevent Fraud Offence

The cases we are concerned with are unlikely to be adequately addressed by the new failure to prevent economic crime offence in the Act. The idea is that an organisation would be guilty of the failure to prevent offence on a strict liability basis when someone associated with the company commits an economic crime (like fraud) *unless* the organisation can show that it had such preventive procedures in place as would be reasonable under the circumstances. This amounts to a negligence standard for imposing liability. Consider the provision creating this new offence:

**199 Failure to prevent fraud**

(1) A relevant body which is a large organisation … is guilty of an offence if … a person who is associated with the body … commits a fraud offence intending to benefit (whether directly or indirectly)—(a) the relevant body, or [relevant others] …

(4) It is a defence for the relevant body to prove that, at the time the fraud offence was committed—

(a) the body had in place such prevention procedures as it was reasonable in all the circumstances to expect the body to have in place, or

(b) it was not reasonable in all the circumstances to expect the body to have any prevention procedures in place.^41^

This provision will not generate criminal liability for the organisation in Separate Knowledge (Culpable Interference). The reason is simple: in that case, there was actually no complete crime of fraud or false statement (or other relevant economic crime) that was committed by any individual. But this is required by the failure to prevent model. The organisation will be inculpated only if an individual committed the whole offence. That is not what we have in the hypothetical we are considering because no individual had the required *mens rea* of awareness of the (risk of) falsehood in the report. So, this failure to prevent offence will not produce a conviction for the company in Separate Knowledge (Culpable Interference). The underlying trouble is that the failure to prevent model remains committed to the Individualist Constraint.

### B. Solution 2: Expanding the Identification Doctrine

One might think the Act’s expanded identification doctrine has better prospects for dealing with the problem cases we are concerned with. The idea is that economic offences like fraud that were committed by a *senior manager* (with the requisite *mens rea*, etc) would be attributed to the organisation. The relevant statutory text (similar to the Law Commission’s proposal, though without the ‘consent or connivance’ language^42^) is this:

**196 Attributing criminal liability for economic crimes to certain bodies**

(1) If a senior manager of a body corporate or partnership (“the organisation”) acting within the actual or apparent scope of their authority commits a relevant offence … the organisation is also guilty of the offence …

(2) “Relevant offence” means an act which constitutes—

(a) an offence listed in Schedule 12 (“a listed offence”),
(b) an attempt or conspiracy to commit a listed offence,
(c) an offence—
  (i) under Part 2 of the Serious Crime Act 2007 (England and Wales and Northern Ireland: encouraging or assisting crime) in relation to a listed offence, or …
(d) aiding, abetting, counselling or procuring the commission of a listed offence.^43^

Would this expanded identification doctrine adequately address cases like Separate Knowledge (Culpable Interference)? One might initially think not because an individual (a senior manager) still needs to commit the crime and have the *mens rea* for the offence—eg some awareness of the falsehood in the report (that it is or may be false^44^). But that did not happen in Separate Knowledge (Culpable Interference). Neither Alice nor Betty, nor any senior manager, had the *mens rea* for fraud. Since the expanded identification doctrine still appears committed to the Individualist Constraint, it may seem not to capture cases like Separate Knowledge (Culpable Interference).^45^

However, this is too quick. One predicate offence in sub-section (2)(b) speaks to our precise scenario: section 46 of the Serious Crime Act (SCA) 2007. It offers the best prospect of conviction in the scenarios we are concerned with. The text is this:

**[section 46] Encouraging or assisting offences believing one or more will be committed**

(1) A person commits an offence if—

(a) he does an act capable of encouraging or assisting the commission of one or more of a number of offences; and
(b) he believes—
  (i) that one or more of those offences will be committed (but has no belief as to which); and
  (ii) that his act will encourage or assist the commission of one or more of them.

(2) It is immaterial for the purposes of subsection (1)(b)(ii) whether the person has any belief as to which offence will be encouraged or assisted.^46^

This offence is relevant to Separate Knowledge (Culpable Interference) because senior management committed acts that it believes will encourage a wide range of offences through their interference with the proper flow of information within the company (ie preventing Alice and Betty’s teams from talking, etc), while believing that some of these offences will be committed—though they do not know precisely which ones. Nonetheless, while some versions of our case would fall within section 46, I will argue that *there are some that very likely would not be caught by section 46*. That is, the scenario sketched in section 2 above must be disambiguated, and if the facts are specified in certain plausible ways, a conviction would be unlikely under section 46 for any individual senior manager and thus for the company under the extended identification doctrine. This will reveal the core instance of the problem I am arguing corporate criminal law does not adequately address even after the 2023 Act. (We will return to the complications of section 47 below.)

Begin with the version of the case that likely *would* be caught by section 46:

Version 1 (Superficial Management): Consider a version of Separate Knowledge (Culpable Interference) where management does not think too carefully about matters. They come to believe (perhaps inaccurately) that their acts of interference will encourage others to commit crimes, including fraud, and believe *at least in general terms* that some such crimes will be committed.

In this version of the case, where senior management reasons only in this superficial way, the section 46 offence is committed and it can be attributed to the company.

However, there are more culpable and worrisome versions of the case, which likewise involve interference with the flow of information but where management is sneakier and thinks about matters in a more sophisticated, legally accurate way. This is the central instance of the scenario that section 46, and hence the expanded identification doctrine, fails to capture.

Version 2 (Sneaky Managers): Consider a version of Separate Knowledge (Culpable Interference) where members of senior management, including Sam (who is just one representative example), interfere with the proper flow of information within the company in ways they believe (at least in the abstract) very likely will assist the *actus reus* of multiple offences, including fraud (related to falsehoods in the company’s reports, perhaps, or other public statements).^47^ But Sam also believes his interventions will prevent any individual from actually forming the *mens rea* for fraud—ie prevent anyone from obtaining any awareness of these falsehoods, if any exist. He believes his actions (like splitting up various teams in the company and giving them confusing instructions) will make it more likely that someone will make some kind of false statement in some context or other, but at most unwittingly. Sam himself has no specific suspicions or doubts about anything in the reports the company issues, as he is only aware of their existence in general and in passing. (The same is true of the other managers, who hold the same beliefs about their own acts of interference in the information flow within the company. Sam is but a representative example.)

Here, it is likely that neither Sam nor any other senior manager has committed the section 46 offence. If the manager in question has thought about matters this carefully and (as is plausible) does not believe any individual will form the *mens rea* of the offence, then it is not the case that he believed ‘that one or more of those offences will be committed’ (section 46(1)(b)(i)). Accordingly, it seems liability under section 46 would not be forthcoming.

There are two additional doctrinal wrinkles that must be addressed to more conclusively show that the illustrative senior manager in Version 2, Sam, does not have the *mens rea* for the section 46 offence. I will argue that these are not viable routes to liability. However, dealing with them is necessary to bolster the conclusion that section 46 does not capture our core case of informational interference by management.

The first wrinkle is that section 46 is actually an exceptionally broad offence because of the additional ways one can be guilty of it specified in section 47(5). Still, in Version 2, Sam (our representative manager) does not satisfy section 47(5) and would still elude conviction. The text:

**[section 47(5)]** In proving for the purposes of this section whether an act is one which, if done, would amount to the commission of an offence—

(a) if the offence is one requiring proof of fault, it must be proved that—

(i) D believed that, were the act to be done, it would be done with that fault;
(ii) D was reckless as to whether or not it would be done with that fault; or
(iii) D’s state of mind was such that, were he to do it, it would be done with that fault;^48^

We already addressed (a)(i). As noted, Sam does not believe that the offence of fraud will be done with the ‘fault’ (*mens rea*) required for fraud (knowledge that the statement is or may be false).

The same reasoning shows that he is not reckless as to the primary actor’s *mens rea* as required under (a)(ii). Because Sam does not think it likely that anyone will become *aware* that they are or may be making false statements (ie discover the statements in the report that are or may be false), Sam does not qualify under section 47(5)(a)(ii) as *reckless* as to the fact that anyone would do the *actus reus* with the required *mens rea*. Sam might well believe, perhaps overconfidently, that the company employees will not become aware of any falsehoods in the report—and perhaps specifically because of the efforts of senior management.

Finally, Sam does not commit the offence under (a)(iii). After all, Sam also does not himself have the fault required for fraud. He is stipulated not to know about the specific report in question or its contents—just that reports generally issue from the teams he has interfered with. Therefore, Sam does not qualify under section 47(5)(a)(iii) either: were he to do the *actus reus* for fraud himself (ie make the relevant statement in the report), he would not be doing the *actus reus* with the required *mens rea* for the offence (ie knowledge that this statement is or might be false).

Thus, we reach the following preliminary conclusion. Sam, like the other managers, in Version 2 (Sneaky Managers) likely will not be guilty of the section 46 offence, so the company also would not be convicted under the extended identification doctrine. However, this seems perverse, as Sam’s effort to avoid liability for himself and the company by preventing others from forming the *mens rea* of other offences does seem highly evasive and thus culpable.

This leaves the second wrinkle. This part of SCA 2007 contains another extremely broad provision, namely section 47(4), and it is unclear how courts seeking to understand it in good faith would apply it to Sam in Version 2 (Sneaky Managers). It is conceivable, if unlikely, that section 47(4) might be construed to render Sam guilty of the section 46 offence. The relevant text:

**[section 47(4)]** If it is alleged under section 46(1)(b) that a person (D) believed that one or more of a number of offences would be committed and that his act would encourage or assist the commission of one or more of them, it is sufficient to prove that he believed—

(a) that one or more of a number of acts would be done which would amount to the commission of one or more of those offences; and
(b) that his act would encourage or assist the doing of one or more of those acts.^49^

Could Sam be found guilty of the section 46 offence with the benefit of this provision? I think it is unlikely, though I want to be cautious because I suspect courts applying it will struggle with its vagueness and complexity. For one thing, the provision states ‘it is sufficient to prove …’, but sufficient for what, one wonders. An outright conviction with nothing else required? That seems unlikely. But what other conditions are implied here? It is not clear. Furthermore, sub-section (a) says it is sufficient to prove ‘that one or more of a number of acts would be done which would amount to the commission of one or more of those offences …’. But, again, what counterfactual circumstances are envisioned by the phrase ‘would amount to the commission of one or more of those offences’? That it would amount to one of the reference offences *if the other elements were satisfied*, including its *mens rea*? If so, section 47(4) would be redundant given that we already have section 47(5)(a)(i). But it is unclear how else to read section 47(4).

In short, section 47(4) is genuinely confusing. It is also worth noting that scholarly construals of this provision also do not suggest that Sam would be guilty of the section 46 offence. John Child’s effort to deal with the confusing language of section 47(4) is as plausible as anyone’s, and his proposal is to read in a requirement of recklessness as to the primary actor’s having *mens rea* of the encouraged offence (much like section 47(5)(a)(ii)).^50^ But, as seen in considering the first wrinkle—specifically, section 47(5)(a)(ii)—Sam lacks such recklessness as to the primary actor’s having *mens rea*. Therefore, I doubt that section 47(4) would be a route to section 46 liability for Sam.

Nonetheless, section 47(4) is admittedly sufficiently vague that we cannot be sure what courts will do with it. This points to a general problem with the expanded identification doctrine in the Act: it inherits all the vagueness and potential for overbreadth of the inchoate encouraging offences in SCA 2007, sections 44–6. I suspect this will be one unwelcome result of the expanded identification doctrine as drafted.

If section 47(4) creates uncertainty about Sam’s liability for the section 46 encouraging offence, it is worth noting that this will be somewhat offset by the broad affirmative defence in SCA 2007, section 50(2)(c), which states that ‘[a] person is not guilty of an offence under this Part if he proves … that it was reasonable for him to act as he did in the circumstances as he believed them to be’. This escape valve would allow Sam to construe his actions in as favourable a light as he can manage. With good trial counsel, it is not implausible he could convince a jury to find (perhaps mistakenly) that his conduct was reasonable given the business requirements and professional or personal pressures he faced at the time. Regardless of how one thinks section 50 *should* be applied, normatively speaking, the reality is that the practical effect of section 50 is to reduce the likelihood of convicting Sam for the section 46 offence—perhaps enough to offset any increased likelihood of conviction due to the confusing and vague language in section 47(4).

Therefore, I conclude that the expanded version of the identification doctrine, even incorporating the section 44–6 inchoate offences of SCA 2007, is not likely to be sufficient to capture all the versions of Separate Knowledge (Culpable Interference) that we should be worried about. Particularly, the most sophisticated actors and their companies—as illustrated by Sam in Version 2 (Sneaky Managers)—would still most likely avoid conviction.^51^

## 5. *What* Not *to Do About the Limitations of the Extended Identification Doctrine*

I have argued that the extended identification doctrine does not go far enough. Gaps remain that the criminal law, given its particular condemnatory force,^52^ should fill—ie cases involving culpable efforts to evade the law’s demands. The obvious solution might seem to be to expand section 46 further so it will cover senior managers like Sam in Version 2 (Sneaky Managers). Perhaps section 46 could be expanded to make Sam guilty because (i) he was aware his actions would encourage others to do the *actus reus* of relevant offences and (ii) he was aware his conduct would prevent these actors from forming the requisite *mens rea*.

However, this would be a very worrisome policy, with serious drawbacks. The main problem^53^ is that section 46 is already extremely broad, and has faced much criticism for that reason.^54^ My view is that we should not extend it further, as this risks improperly sweeping in low-culpability actors along with high-culpability actors like Sam. Here is an example where such an expansion to section 46 (ie expanding this encouraging offence to include acts that assist an *actus reus* but block the formation of the *mens rea*) would misfire. It is another version of our central case but involving *justified* interference.

Version 3 (Justified Interference): Suppose management does the same things as in the original Separate Knowledge (Culpable Interference) case, but for good reasons related to legitimate business aims—*not in order to avoid liability*. Suppose management moves Alice and Betty’s teams because of personnel issues (avoiding conflicts between team members), giving a new project to the one team because it is a key opportunity for growing the company’s business and sending confusing instructions because of laborious data protection requirements. But management knows it is statistically very likely that these moves together will lead to the *actus reus* of some crime or other being committed as a result, though they are only aware of a broad range of possible offences (fraud, conspiracy, embezzlement, etc). They believe only that this is statistically likely, though their acts of interference were done for acceptable reasons.

If we expand section 46 in the manner contemplated above, so that Sam is guilty because he was aware he was encouraging the *actus reus* of offences like fraud but preventing others from forming *mens rea*, then we would also have to convict the manager in Version 3, who interfered in the company’s information flow but for good reasons. That would extend liability too far.

Of course, supporters of extending individual liability may reply as follows. They might try to narrow the proposed amendment to section 46 so it focuses only on *culpably* preventing others from forming the *mens rea*—for example, doing so *for the purpose of avoiding liability*. One reason I am not keen on this solution is that it would necessitate a difficult and nebulous inquiry into the nature and quality of the reasons for which management interfered in the proper information flow within the company. To condition liability on a detailed and potentially invasive investigation of what a manager’s personal reasons were for interfering in the flow of information in the company in the ways they did (whether this was *really* to avoid liability or perhaps also for justifiable business reasons, or merely personal reasons like a petty desire to save face or inconvenience an annoying colleague, etc) would render the line between guilt and innocence very difficult to discern *ex ante*. This would undermine the law’s ability to provide fair notice and give actionable guidance—both rule of law problems.

But there is also a deeper problem with this reply (expanding section 46 to cover culpably preventing others from forming the *mens rea* in order to avoid liability). Adjusting existing forms of *individual liability*, which remain committed to the Individualist Constraint, in order to address the present problems seems simply to be the wrong kind of tool for the job. The focus on individual liability, I suggest, is a red herring. Interference in information flow is very often a *structural* problem relating to how the company is organised. As such, I suggest it requires a more distinctively structural, or non-individualist, response.

To clinch the argument, let me illustrate using two examples of structural problems where this sort of individualistic fix—ie a narrow expansion to section 46 which would require preventing others from forming the *mens rea* for the underlying offence *in order to avoid liability*—still will not be sufficient to address the relevant structural problems. These are sub-varieties of Version 2 above, and so incorporate the facts of that case unless otherwise specified. They highlight the difficulties that even such a carefully crafted individualistic approach to liability will have with adequately addressing structural deficiencies in the organisation’s handling of information:

Version 2.1 (Minimal Individual Contribution): Consider a version of the Separate Knowledge case in which the relevant forms of interference by management were carried out by different individual managers, all hoping to help insulate the company a little bit from a wide but underspecified range of legal, regulatory or reputational problems. However, none of these individual managers believes that their *individual contribution* to keeping knowledge separate within the organisation (that individual’s own actions, the particular steps they took or perhaps their vote in favour of a package of such steps) would actually encourage any *actus reus* elements of fraud. No senior manager believes that their individual conduct will make it any more likely that the *actus reus* of fraud or other economic crimes will be committed. This is because each manager believes that the actions of *other managers* will be what creates the substantial interference with the proper information flow within the company. Each manager believes that their own action makes *no contribution* to the *actus reus* of fraud (issuing false statements) or the *actus reus* of other offences. As before, the senior managers also believe that they will prevent anyone from forming the specific *mens rea* of fraud or other relevant economic crimes.

The structure at issue in such a case is one where each senior manager lacks the belief that ‘that his act will encourage or assist the commission of one or more’ *actus reus* of the relevant offences, as would be required by an expanded section 46(1)(b)(ii). This is because, given their awareness of other acts of interference by other managers, each manager believes their conduct makes no difference and provides no encouragement or assistance to the *actus reus* of fraud (or other economic crime). In short, the trouble arises because each manager perceives the case to involve *causal overdetermination*: even without his own act of interference, the manager believes other acts of interference by others will be what encourages or assists the *actus reus* of fraud. Hence, each senior manager would not possess the *mens rea* required by section 46(1)(b)—even under the proposed expansion of the offence to include encouraging others to do the *actus reus* of an economic crime while culpably preventing them from forming the required *mens rea* in order to avoid liability.

A second example where the proposed narrow expansion of section 46 would not suffice is this.

Version 2.2 (Reckless Interference): Suppose that the senior managers’ interference in the proper information flow within the company was only *reckless* and therefore still culpable, although not done *intentionally* as the means to avoiding liability. Suppose, that is, that each manager did not *intend* to help the company avoid liability through their acts of interference in the proper flow of information. They did not interfere *for this reason* (suppose they did it for other not-particularly-laudable reasons), but they were aware of the risk and happy to accept that it might increase the chances of keeping legal problems buried or undetected, and thus keep the company out of legal trouble. (As before, they believe their actions will prevent others in the company from forming the *mens rea* of relevant economic offences.)

In this case, the managerial interference could still be culpable. But it also would not fit within the expanded section 46 contemplated above, requiring *intent to avoid liability*, not mere recklessness thereof.^55^ It is a structural problem related to what sophisticated managers will be aware are the likely (unintended) side effects of their actions. So, in Version 2.2, like Version 2.1, I submit that an individual liability solution is not the right response for problems that are in essence a structural problem with the organisation.

## 6. A Better Solution? Direct Liability for the Organisation

I have argued that further extending individual liability is not a promising response to the limitations of the extended identification doctrine, particularly where the problem is structural or organisational (as in Versions 2.1 and 2.2 of our central case) rather than discrete acts of individual wrongdoing. The underlying difficulty is that the identification doctrine, even in its expanded form, is still committed to the Individualist Constraint. An individual needs to possess the whole *mens rea* (whether for the underlying crime or an inchoate crime) to inculpate the company.

Accordingly, my preferred solution would be to pursue direct liability for the company, rather than expand individual liability for existing offences ever further so that increasingly minute, low-culpability bits of conduct get criminalised. Instead, I propose we reconsider the benefits of aggregating different units of culpability within the company where these are sufficiently closely related and sufficiently culpable in the right way to enable an organisation-level conviction (though not an individual conviction). The collective knowledge doctrine would be particularly attractive if it could be judicially adopted through statutory interpretation, rather than requiring further statutory reforms.^56^ Let me outline the substance of the proposal before considering its availability as an interpretation of the statutory text of the 2023 Act.

The rule I propose is roughly the following (a version of the Restricted Collective Knowledge Doctrine from section 2 adapted for this context):

**Restricted Mental State Aggregation**: A piece of knowledge, K, that is the required *mens rea* of an offence can be imputed to an organisation for purposes of convicting it of that offence—even if no individual within the organisation possessed K themselves—if one or more senior manager of the organisation, acting for the organisation’s benefit and within the scope of their employment,

(i) interfered with the proper flow of information or decision-making procedures of the organisation,

(ii) with the intention or awareness of helping the company avoid criminal liability,^57^ and

(iii) knew, or should have known, that without such interference (in sub-section (i)) they or other employees or agents of the organisation would have been highly likely to possess K.

These conditions are supposed to approximate the conditions in which senior managers’ interference in the proper flow of information within the organisation would be sufficiently culpable to render *the company as culpable* as it would have been if a single senior manager had possessed K and the company acted analogously. This form of ‘equal culpability’, I argue, is necessary to avoid overly expansive aggregation or otherwise unfair convictions.^58^ (I am not wedded to all the details of this formulation—they are illustrative and may be further refined.)

This proposal is not such a radical departure from existing approaches to *mens rea*; it is a natural extension of how courts already expand *mens rea* notions to avoid unfairness. One example is the way we permit an individual’s wilful ignorance—defined as suspecting an inculpatory fact and then deliberately avoiding additional information—to satisfy the knowledge element of an offence.^59^ Another way courts stitch together offence elements, which I revisit below, arises within the traditional identification doctrine when courts combine the DMW’s *mens rea* with the *actus reus* the DMW was aware a lower-level employee was carrying out.^60^ These doctrinal approaches involve courts using their interpretive powers to weave together offence elements to enable deserved convictions when acquittals would be unfair or inconsistent with the outcomes in broadly analogous cases. The narrow collective knowledge doctrine I propose is a natural next step from approaches like these. Indeed, adopting a limited form of aggregation to construct a corporate *mens rea* is in many ways a less extreme step than creatively constructing an individual mental state for purposes of imposing liability. After all, an organisation’s *mens rea* is always a legal construction, while constructing *mens rea* for individual human beings entails a greater risk of departing from the real psychological states to which *mens rea* categories are presumed to correspond.

The narrow form of mental state aggregation I am proposing would enable the construction of an organisational *mens rea* in order to convict the company of fraud in our central cases—Version 2 (Sneaky Managers), Version 2.1 and Version 2.2—although without thereby convicting any individual thereof. This makes sense because no individual actor in these cases—not Sam, Alice or Betty—fulfils all the elements of fraud. Nonetheless, the proposed mental state aggregation would apply here to permit the knowledge required for fraud based on the misstatements in the report—specifically, knowledge of the false statement about emissions levels at the company’s plants (not-P)—to be constructed and imputed to the company itself. Because of Sam’s culpable interference in normal operations with the intention (or willingness) to help the company avoid liability, the proposed rule would allow Alice’s knowledge (P entails Q) and Betty’s knowledge (not-Q) to be combined to draw the inference (not-P) that someone in the company very likely would have reached absent Sam’s culpable interference. Accordingly, under the proposed rule, the company could be charged with knowledge of the false statement in the report, and directly convicted of a fraud offence, in the narrow circumstances of cases like Version 2 (Sneaky Managers), Version 2.1 or Version 2.2. But no individual would similarly face conviction for fraud or encouraging fraud (nor should they). In this way, the proposed rule has the normative advantage of constituting a direct legal response at the level of organisations to the structural problems at issue in these problem cases, rather than seeking to stretch individual liability even further in a very likely futile effort to address structural forms of fault.

The key remaining question, however, is whether this narrow form of mental state aggregation really is available as an interpretation of the expanded identification doctrine in the 2023 Act. Recall the language in section 196:

(1) If a senior manager of a body corporate or partnership (‘the organisation’) acting within the actual or apparent scope of their authority commits a relevant offence after this section comes into force, the organisation is also guilty of the offence.^61^

The conclusion I will argue for is a conditional one: there are doctrinal reasons against a very strict interpretation of this text and in favour of a wider reading that would license a constrained collective knowledge doctrine, but the likelihood of this wider reading being adopted depends on how purposivist in orientation the courts are—a matter I cannot resolve here. Therefore, my aim here is just to lay out the case for the wider reading, which would permit employing the collective knowledge doctrine. Litigators will have to assess its chances of success on the ground, but I suggest it is a theory that prosecutors may find worthwhile to test in court. My remaining argument in this section has two parts. First, I argue that while a very strict construction of section 196 would not permit the sort of mental state aggregation proposed here, this interpretation leads to problematic legal results that conflict with existing identification doctrine precedent. However, on a broader reading of the Act’s language, restricted mental state aggregation would form part of an available interpretation. Consider each interpretation in turn.

### A. The Strict Interpretation

On a strict interpretation, section 196 would permit the organisation to be convicted if and only if a senior manager commits the full crime herself—performs the *actus reus* and possesses the *mens rea*. This is suggested by the phrase ‘commits a relevant offence’. The literal reading of this phrase is that it refers to a whole, complete offence. This strict reading, of course, rules out adoption of the restricted collective knowledge doctrine I have proposed.

This strict interpretation faces problems, however. Specifically, it would conflict with existing identification doctrine precedent. The identification doctrine case law suggests that while the *mens rea* must be possessed by a DMW, the *actus reus* could also be carried out independently by a lower-level employee. That is, the existing identification doctrine allows a company to be inculpated not only whenever a single DMW commits the full offence, but also in certain additional cases: namely, where the *actus reus* was done by *another* employee than the DMW who had *mens rea* and this *actus reus* flows from the DMW in the right way, such as if the DMW ordered or enabled the *actus reus* or the DMW was aware of the *actus reus* and did not intervene to stop it.^62^ Suppose a low-level employee commits the *actus reus* of fraud (makes misleading representations) but a board member who counts as a DMW is aware of the *actus reus* and the DMW has the requisite *mens rea*—knowledge of the (risk of) falsity of the representation that the low-level employee makes and intent thereby to make a gain in money or property—but the DMW does nothing to intervene and block the *actus reus* from being carried out. Here, the existing identification doctrine would likely permit conviction of the organisation.

I offer three considerations in support of this view of the existing identification doctrine, what Dsouza calls the *split-identification model*,^63^ which allows the *mens rea* and *actus reus* elements to be fulfilled by different people in the company. First, *HL Bolton (Engineering) Co v TJ Graham & Sons* suggests that different people can act and think for the corporation:

A company … has a brain and nerve centre which controls what it does. It also has hands which hold the tools and act in accordance with directions from the centre. Some … in the company are mere servants and agents who are nothing more than hands to do the work … Others are directors and managers who represent the directing mind and will of the company, and control what it does. The state of mind of these managers is the state of mind of the company and is treated by the law as such.^64^

Second, *Tesco* was itself a case where split identification is relevant.^65^ There, a sales assistant performed the *actus reus* of putting out packs marked with a price that contradicted the adverts in the store, but it was the store manager who was allegedly negligent in failing to ensure (as was his responsibility) that the prices charged matched the daily special offers. Liability for the company was blocked because the store manager was not a DMW. But there was no indication in the judgment that if the store manager *had* been a DMW there would be any hurdle to convicting Tesco on the ground that a different employee brought about the offence’s *actus reus* than possessed its *mens rea*.

Third, *El-Ajou v Dollar Land Holdings* suggests that it is the *mens rea* of the DMW that really matters for convicting the company, and the act elements of the offence can be performed by others. The judgment states:

Once [the DMW’s] knowledge is treated as being the knowledge of the company in relation to a given transaction … the company continues to be affected with that knowledge for any subsequent stages of the same transaction. So … if … [the DMW] had resigned or died a week earlier, I do not think that the DLH could have said that it received the money without imputed knowledge of the fraud.^66^

Indeed, the Law Commission similarly recognised that the *actus reus* need not always be completed by a DMW herself who has *mens rea*.^67^

Accordingly, the existing identification doctrine is naturally understood as the split-identification model suggests.^68^ Cases will frequently arise where a senior manager is aware of lower-level employees carrying out the *actus reus* of an offence and doing nothing to stop it (thereby tacitly approving it) despite this manager herself possessing the requisite *mens rea* (or coming close by being reckless). It is open to question whether Parliament, in passing the 2023 Act, intended (if they considered it at all) to countermand this existing identification doctrine case law, which allows the offence to be attributed to the company where the DMW (now senior manager) possesses the *mens rea* but another low-level employee independently does the *actus reus* without *mens rea*. This is a substantial problem for the strict interpretation of the statutory text we are considering.

One might object that this is a non-issue because the actor in such cases still might be convicted of an encouraging offence under SCA 2007, sections 44–6, which then could be attributed to the company. However, a conviction under these sections would be unlikely even if read in conjunction with section 47(5)(a)(iii), which says it is enough if the encouraging actor herself has the *mens rea* for the underlying offence. The reason is that the manager in question here actually commits no affirmative act of encouragement that she believes to assist the complete crime; at most, she omits to intervene to prevent the *actus reus* from being committed. Thus, she would not satisfy section 45(a) or 45(b)(ii), or the analogous elements of the section 46 offence (namely sections 46(1)(a) and 46(1)(b)(ii)). Furthermore, she can always attempt to invoke the section 50 affirmative defence that her conduct was reasonable under all the circumstances, especially by highlighting any sympathetic aspects of her situation. At least, this injects uncertainty in such cases, since the contours of reasonableness remain unclear. Furthermore, it would be a labelling problem to convict the company of an inchoate offence of, say, encouraging fraud when the company collectively committed *fraud* and the traditional identification doctrine under a split-identification model would permit a fraud conviction. Thus, relying on the inchoate section 44–6 offences is unlikely to avoid the present problem.

The upshot is that the strict reading of the proposed statutory text would significantly narrow the scope of the identification doctrine by requiring that a senior manager commit the *whole* offence—both do the *actus reus* and possess the *mens rea*—in order for the offence to be attributed to the company. This goes against existing identification doctrine case law, which permits the *actus reus* to be independently performed by a different actor in the company than the DMW who possesses the *mens rea*. It is unlikely Parliament would have intended this narrowing of the identification doctrine when the advertised purpose of the Act’s reforms is to bring new actors—senior managers—within the scope of the identification doctrine.^69^

### B. A Wider Interpretation

There is also a wider interpretation of the Act’s text, which avoids this problem. Moreover, this reading is compatible with adopting a restricted version of the collective knowledge doctrine. This wider interpretation would take the phrase referring to a senior manager who ‘commits a relevant offence’ to mean not that this manager must do the *actus reus* and possess the full *mens rea* herself, but rather that, for purposes of this statute, she must ‘commit the offence’ either herself or *through, or in concert with*, others in the organisation. On this wider reading, ‘commits a relevant offence’ would be read to mean ‘commits the full offence herself or causes, procures or orchestrates the commission of its elements by others in the organisation’. On this reading, *mens rea* is still required for convicting the organisation of a given offence, but this *mens rea* need not be possessed in full within one individual senior manager. It would be enough if the *mens rea* could be aggregated from among the mental states of a collection of senior managers or others to whom they have delegated their responsibilities (much the way split identification aggregates a single person’s *mens rea* with another person’s *actus reus*).

This wider reading would have two important implications. First, a senior manager could ‘commit’ a relevant offence for purposes of this statute without herself doing the *actus reus*. It would be enough if she induced or orchestrated its commission by others. Second, on this reading, the specific individual manager would not herself have to possess the full *mens rea* of the offence (say, fraud) to count as ‘committing’ it for purposes of this statute and thereby inculpate the company. Hence, the senior manager would not have to be convictable of the offence herself for it to be attributable to the company.

Instead, the key move in this wider reading is to allow *commission* of an offence, for purposes of the statute, to involve procuring or orchestrating the commission of the various offence elements by a group of others in the organisation. Hence, the *mens rea* for an offence could be constructed and imputed to the organisation through aggregation in response to culpable interference by senior management that functioned to prevent the full *mens rea* from being formed by any single individual. A senior manager could *commit* an offence, for purposes of the statute, when the *mens rea* for the offence is to be found distributed across several individuals *because* of the senior manager’s culpable steps to prevent the full *mens rea* being formed by any particular individual. Here, mental state aggregation would operate to negate the senior manager’s culpable interference in the organisation’s information-sharing processes—processes that without such interference would have led an individual to possess the full *mens rea*. In that case, the *mens rea* would be deemed to be possessed by the organisation, even if no individual can be found who possessed the full *mens rea*. On the wider reading, such orchestration of the offence elements across several individuals would count as the manager committing the offence.

This admittedly is a bolder interpretation of the statutory language, which strict textualist courts may be uncomfortable with. But courts interested in reading statutes more capaciously in service of good policy, and to avoid overturning precedent that Parliament likely did not intend to countermand, may well prefer this wider interpretation. It avoids the problematic implications of the strict reading mentioned above, namely ruling out split identification suggested in current case law. Instead, the wider interpretation is a natural expansion of the common law in line with legislative changes that seek to move beyond a single directing mind and will. The statute’s broader focus on senior managers opens the door to the wider interpretation. It is a suitable response—premised on direct corporate liability rather than implausibly broad forms of individual liability—to the problem of senior management interfering with the proper flow of information and decision making within an organisation to suppress potentially damaging information. The wider interpretation of the statutory text—which allows senior managers to commit offences through a group of others who together (if not individually) satisfy the offence elements—would be a judicially available basis for limited mental state aggregation.

The question of whether the courts are sufficiently policy-oriented or purposivist in orientation to adopt this wider interpretation of section 196 is an issue I cannot settle here. I would suggest the possibility is not foreclosed,^70^ but I offer only a conditional conclusion here: assuming courts are willing to interpret the 2023 Act’s provisions in light of the broader aim of this legislation to expand the scope of corporate criminal liability (not narrow it the way the strict interpretation would), then there are substantial reasons to favour the broader interpretation of section 196 I have suggested. It would not only avoid the doctrinal costs of the very strict interpretation, but would secure the benefits of the collective knowledge doctrine without the dangers of expansive forms of individual liability that might otherwise be a tempting response to the cases in question. The collective knowledge doctrine, after all, is a more narrowly tailored tool for solving the present problems of informational abuse in organisations than wider expansions to individual liability would be.

One might object to the wider interpretation because the Court of Appeal in *Attorney General’s Reference (No 2 of 1999)* said they ‘reject the suggestion that aggregation has any proper role to play’.^71^ Nonetheless, this case is distinguishable, and its reasoning on this point no longer persuasive. The case was about convicting a corporation of gross negligence manslaughter under the old identification doctrine, using aggregation of management failings to find gross negligence. However, this case was decided before the Corporate Manslaughter and Corporate Homicide Act 2007 opened up the gross negligence inquiry to permit aggregation. That Act approves aggregation in contemplating that the jury may ‘consider [whether] there were attitudes, policies, systems or accepted practices within the organisation that were likely to have encouraged any such failure … or … produced tolerance of it’.^72^ Further, *Attorney General’s Reference (No 2 of 1999)* was decided under the old identification doctrine that was cabined only to the mental state of the company’s DMW, which is far narrower than the expanded version in the 2023 Act. The expanded identification doctrine permits any number of senior managers to inculpate the company, which opens the door to aggregation. Thus, the Court of Appeal’s claim in this case that ‘[t]he case against a corporation can only be made by evidence … showing guilt on the part of the corporation as such’^73^ is compatible with aggregation as the company is now identified with a wider class of individuals than just a singular DMW.

## 7. Conclusion

I have argued that a restricted collective knowledge doctrine is warranted in response to certain shortcomings of the expanded identification doctrine in the Economic Crime and Corporate Transparency Act 2023. We saw that the expanded identification doctrine, despite inheriting the full sweep (not to mention complexity and confusion) of the inchoate encouraging offences in the Serious Crime Act 2007, sections 44–6, still does not go far enough. It is unlikely to allow convictions in response to culpable senior managers who interfere in the proper flow of information within the company in order to obtain the benefit of the *actus reus* of economic crimes, while seeking (or accepting) the evasion of liability by preventing others in the organisation from forming the *mens rea* of those crimes. A criminal law response is called for to these cases (of which we saw multiple examples, particularly Versions 2, 2.1 and 2.2), not just a regulatory or private law response, since they involved conscious evasion of the law that merits the distinctive expressive condemnatory force of the criminal law.^74^ To adequately deal with these scenarios, I argued that a restricted form of the collective knowledge doctrine is a promising solution.

Furthermore, I argued that this approach—dubbed Restricted Mental State Aggregation—could be imposed through judicial interpretation, which has the practical advantage of not requiring further statutory amendments. Admittedly, a strict construction of the language expanding the identification doctrine in section 196 of the 2023 Act would not sit comfortably with my proposed form of mental state aggregation. However, I suggested that this narrow reading has doctrinal difficulties, which in turn supports a wider reading that is compatible with limited mental state aggregation. Accordingly, I offered a conditional conclusion: if courts are sufficiently purposivist in orientation to be willing to interpret the 2023 Act in light of its broader aim of expanding the scope of corporate criminal liability (not narrow it as the strict interpretation would), then there are substantial reasons to favour a broader interpretation of section 196, which would permit limited mental state aggregation.

It remains unclear how courts will construe the 2023 Act’s language expanding the identification doctrine. But I submit that the restricted version of the collective knowledge doctrine defended here would be worth keeping in the prosecutor’s toolkit and pursuing in court through test litigation.

## Footnotes

1 For a useful summary, see Law Commission, *Corporate Criminal Liability: An Options Paper* (2022) para 3.56–3.72 (Options Paper) <www.lawcom.gov.uk/project/corporate-criminal-liability/>; see also chs 3–4.

2 Royal Assent was given on 26 October 2023. See Press Release, 26 October 2023 <www.gov.uk/government/news/robust-new-laws-to-fight-corruption-money-laundering-and-fraud>.

3 See Press Release, 15 June 2023 <www.gov.uk/government/news/more-action-to-fight-fraud-bribery-and-other-economic-crime>. For the offences covered by the expanded identification doctrine, see Sch 12 of the 2023 Act <www.legislation.gov.uk/ukpga/2023/56/schedule/12/enacted>. The government indicated an ambition to similarly expand the identification doctrine to all criminal offences. Policy Paper on Identification Principle for Economic Crimes, 1 March 2024 <www.gov.uk/government/publications/economic-crime-and-corporate-transparency-act-2023-factsheets/economic-crime-and-corporate-transparency-act-identification-principle-for-economic-crime-offences> (discussing the government’s Economic Crime Plan 2 and Fraud Strategy).

4 Options Paper (n 1).

5 For example, the Crown Prosecution Service (CPS), Serious Fraud Office (SFO) and other stakeholders were supportive. See Press Release, 26 October 2023 <www.gov.uk/government/news/robust-new-laws-to-fight-corruption-money-laundering-and-fraud> (quoting the CPS and SFO directors as welcoming the new legislation); The Law Society Statement, 27 February 2024 <www.lawsociety.org.uk/topics/anti-money-laundering/economic-crime-and-corporate-transparency-act> (welcoming 2023 Act and its aims); Linklaters, BusinessCrime Links, 27 October 2023 <www.linklaters.com/en/insights/blogs/businesscrimelinks/2023/october/the-economic-crime-and-corporate-transparency-act-receives-royal-assent> (discussing views of this legislation as a ‘game-changer’).

6 See nn 15 and 18.

7 See Options Paper (n 1) para 3.81 (emphasis in original).

8
*United States v Bank of New England* 821 F.2d 844, 856 (1st Cir 1987); Mihailis Diamantis, ‘Functional Corporate Knowledge’ (2019) 61 William & Mary Law Review 319, 344. For more comprehensive discussion of sources examining this doctrine, see n 10 and nn 16–21.

9 See n 15.

10 See Alex Sarch, *Criminally Ignorant: Why the Law Pretends We Know What We Don’t* (OUP 2019) ch 9.

11 I cannot undertake here the important additional task of empirically demonstrating the pervasiveness of these problem cases. That will have to be left to subsequent work. Note that the government’s cautious impact assessment of the expanded identification doctrine, suggesting it would impact 0–3 cases per year, is *not* a decisive reason to think the problem cases this article confronts are unimportant. See ‘Impact Assessment: Reform of the Identification Doctrine’ (13 June 2023) para 78 <https://assets.publishing.service.gov.uk/media/648c20455f7bb7000c7fabca/5_IDD_Impact_Assessment.pdf>. After all, if the problem cases this article is concerned with are not captured by existing criminal law doctrine, then they are unlikely to be prosecuted and hence counted in this impact assessment. If the broader interpretation of the Act that Section 6 below proposes is pursued, this would enhance the impact of this legislation.

12 David Ormerod and Rudi Fortson, ‘Serious Crime Act 2007: The Part 2 Offences’ [2009] Crim LR 389, 397.

13 This is a revised version of a case I have discussed previously. See Sarch (n 10) ch 9. Although hypothetical, it draws on features from real cases. See *United States v TIME-DC, Inc* 381 F Supp 730 (WD Va 1974); *United States v Bank of New England* 821 F.2d 844 (1st Cir 1987). The Law Commission considers a case that is similar in structure. Options Paper (n 1) para 6.44–6.45. However, the details of the Law Commission’s case admit of many interpretations—some culpable, some not. The view I argue for here is ultimately compatible with the Law Commission’s position on mental state aggregation. See n 22.

14 This article relies heavily on hypotheticals because it makes the abstract doctrines discussed more vivid and concrete, thus facilitating careful analysis. Using hypotheticals also avoids the factual uncertainty surrounding most real examples of complex corporate misconduct.

15 See *First Equity Corp of Florida v Standard & Poor’s Corp* 690 F Supp 256, 259–60 (SDNY 1988) (‘plaintiffs seek to attribute to Standard & Poor’s the combined knowledge of several of its employees in order to hold the corporation liable for fraud. But plaintiffs’ theory is flawed … A corporation can be held to have a particular state of mind only when that state of mind is possessed by a single individual’); *McGee v Sentinel Offender Servs, LLC*, 719 F.3d 1236 (11th Cir 2013) (noting that other circuits had encountered ‘attempt[s] to weave the fragmented actions of various agents into corporate *mens rea*’, but ‘[w]hatever the theory’s status in other jurisdictions … we decline to recognize it here’); *United States v Sci Applications Int’l Corp* 626 F.3d 1257, 1274 (DC Cir 2010) (expressing ‘skepticism about corporate intent theories that rely on aggregating the states of mind of multiple individuals’).

16
*United States v Bank of New England* 821 F.2d 844, 856 (1st Cir 1987) (‘A corporation cannot plead innocence by asserting that the information obtained by several employees was not acquired by any one individual who then would have comprehended its full import. Rather the corporation is considered to have acquired the collective knowledge of its employees and is held responsible for their failure to act accordingly. Since the Bank had the compartmentalized structure common to all large corporations, the court’s collective knowledge instruction was not only proper but necessary’). However, some have argued that *Bank of New England* adopted the collective knowledge doctrine not as a substantive liability rule, but as an evidential rule which permits an *inference* of individual knowledge from the collection of mental states possessed by multiple employees. See *United States v Sci Applications Int’l Corp* 626 F.3d 1257, 1274 (DC Cir 2010). For civil cases applying the collective knowledge doctrine as a pleading rule, see *Teamsters Local 445 Freight Div. Pension Fund v Dynex Capital Inc* 531 F.3d 190 (2nd Cir 2008); *Makor Issues & Rights, Ltd v Tellabs Inc* 513 F.3d 702, 710 (7th Cir 2008).

17 Eli Lederman, ‘Models for Imposing Corporate Criminal Liability: From Adaptation and Imitation Toward Aggregation and the Search for Self-Identity’ (2000) 4 Buffalo Criminal Law Review 641, 654–5.

18 The term ‘Individualist Constraint’ is mine, but the requirement is familiar. See the cases cited in n 15; Lederman (n 17) 667; Diamantis (n 8) 340.

19 Sarch (n 10) 243–60.

20 The nature of this benefit will vary from case to case. In fraud or market abuse scenarios, say, they might involve misleading a victim into taking investment actions, entering into or exercising rights under a contract, or offering to do the same, in ways that ultimately benefit the company.

21 Sarch (n 10); for a distinct but largely compatible proposal, see Diamantis (n 8).

22 The Law Commission’s Options Paper does not close the door to mental state aggregation completely. See Options Paper (n 1) paras 6.44–6.45. The Commission acknowledges that ‘[i]t is possible to have aggregate models of corporate knowledge which reflect the fact that knowledge within an organisation might be dispersed’ (para 6.44). Although it considers one example where they deem aggregation to be ‘questionable’ (para 6.45), this is an example of *innocent* separation of knowledge, where there is no culpable interference in proper information flow within the company—and where I would not support aggregation either. I contend only that aggregation is appropriate in response to seriously culpable instances of such interference by senior management. Therefore, my proposal appears consistent with the Law Commission’s position.

23 This is recognised by the Law Commission. Options Paper (n 1) para 3.84; see also n 59. As authority for this proposition in US federal law, see *United States v St Michael’s Credit Union* 880 F.2d 579, 583–6 (1st Cir 1989) (finding sufficient evidence for convicting an individual under wilful ignorance jury instructions for violating Internal Revenue Service filing requirements and imputing this knowledge to the corporation that employed her, which was convicted of the same offences—although the convictions were vacated on other grounds).

24
*Global-Tech Appliances, Inc v SEB SA* 131 S.Ct. 2060, 2070 (2011) (defining wilful ignorance as requiring that ‘(1) the defendant must subjectively believe that there is a high probability that a fact exists and (2) the defendant must take deliberate actions to avoid learning of that fact’). For English wilful ignorance cases, see n 59.

25 That this will not suffice for wilful ignorance can be seen from considering the lower mental state of recklessness. Because wilful ignorance requires belief in a high probability of an inculpatory fact, wilfully ignorant actors are always at least reckless, and recklessness requires at least somewhat particularised awareness of the relevant risk of the fact at issue. Model Penal Code, § 2.02 (One ‘acts recklessly with respect to a material element … when he consciously disregards a substantial and unjustifiable risk that *the material element exists or will result* from his conduct’ (emphasis added)); *People v Hall* 999 P.2d 207, 220 (Colo 2000) (‘recklessness requires that [one] consciously disregard a substantial and unjustifiable risk *of a particular result*’) (emphasis added)); *United States v Mohsin* 904 F.3d 580, 585 (7th Cir 2018) (holding as to sentencing that the evidence ‘does not support [that the defendant] had any awareness the synthetic marijuana products she sold posed a risk of death or serious bodily injury. *General awareness of the health risks of taking medications does not demonstrate a defendant’s specific knowledge or awareness of the risks presented by a particular substance*’ (emphasis added)). See also *In re Refco Inc Sec Litig* No 08 CIV 3065 JSR, 2012 WL 3126834, *3 (SDNY 30 July 2012) (‘vague suspicions [of red flags] are far removed from reckless disregard, let alone actual knowledge’).

26 See Sarch (n 10) ch 9.

27 See Alan Norrie, *Crime, Reason and History* (CUP 2014) 117–19 (discussing the historically important commitment to an individualist approach to criminal liability, also in the UK); see also Celia Wells, *Corporations and Criminal Liability* (2nd edn, OUP 2001) 63–4 (discussing the ‘individualist assumptions which underlie blame attribution’ and observing that these are ‘ill-suited for … organizations’), 75–6 (discussing how the ‘individualist notions implicit in discussions of criminal liability’ are rooted in ‘Western moral thought’).

28 [1972] AC 153, 170 (Lord Reid).

29 [1972] 1 WLR 118.

30
*Tesco* (n 28) 171.

31 ibid 187–8.

32 [2018] EWHC 3055 (QB).

33 Mark Dsouza, ‘The Corporate Agent in Criminal Law—An Argument for Comprehensive Identification’ (2020) 79 CLJ 91, 97.

34 See n 62 and accompanying text.

35 See Options Paper (n 1) para 358 (discussing how the traditional identification doctrine ‘ma[de] it too difficult to convict companies for offences committed for their benefit’); see generally paras 3.56–3.72; James Gobert, ‘Corporate Criminal Liability: Four Models of Fault’ (1994) 14 LS 393.

36 Robin Lööf, ‘Corporate Agency and White Collar Crime—an Experience-Led Case for Causation-Based Corporate Liability for Criminal Harms’ [2020] Crim LR 275, 276.

37 Policy Paper on Identification Principle for Economic Crimes (n 3); Policy Paper on Failure to Prevent Fraud Offence, 1 March 2024 <www.gov.uk/government/publications/economic-crime-and-corporate-transparency-act-2023-factsheets/economic-crime-and-corporate-transparency-act-failure-to-prevent-fraud-offence>.

38 For the jurisdictional extent of the 2023 Act, see ss 196(3) and 218 for geographical limits on convicting the organisation under the expanded identification doctrine.

39 2023 Act s 219(1).

40 ibid s 219(3)(a).

41 ibid s 199; cf the Law Commission’s proposal for such an offence, Options Paper (n 1) paras 8.105–8.106.

42 Options Paper (n 1) paras 4.76–4.77.

43 2023 Act, s 196.

44 Fraud Act 2006, s 2(2)(b).

45 As an anonymous reviewer points out, significant ambiguity remains in the statutory definition of ‘senior manager’. See s 196(4) of the 2023 Act. It is not clear what is meant by an individual having ‘a substantial role’ in making decisions about ‘how a substantial portion of the activities of the organisation are managed or organised’. Still, I set aside this complication here as it pertains to a factual question for the jury. For clarity, I assume the relevant actors in the examples discussed here are senior managers.

46 Serious Crime Act 2007, s 46.

47 Assume there is no agreement between Sam and the other managers that might implicate any conspiracy.

48 Serious Crime Act 2007, s 47.

49 ibid.

50 JJ Child, ‘Exploring the Mens Rea Requirements of the Serious Crime Act 2007 Assisting and Encouraging Offences’ (2012) 76 JCL 220, 225.

51 One might wonder if, for all I have said, it might still be plausible to convict Sam in Version 2 (Sneaky Managers) of fraud on an ‘innocent agency’ theory. This is in play since s 196(2)(d) of the 2023 Act expressly brings aiding, abetting, counseling or procuring a listed offence within the scope of the identification doctrine. (Thanks to Mihailis Diamantis and Mark Dsouza for helpful discussion.) The innocent agency theory holds that if A deliberately uses another person who lacks *mens rea* (or has a personal defence) to commit a given crime, A can be convicted of that crime in much the same way as if A had aided and abetted that crime. For example, in *R v Michael* (1840) 9 C&P 356, the defendant bought a bottle of laudanum and sought to kill her child with it by instructing the person caring for the child to give it a teaspoon of the substance each night. The carer refused to do so and put the bottle on the mantle, where another child found it and fed it to the baby—with fatal results. On appeal, Michael’s murder conviction was sustained, in effect combining Michael’s *mens rea* with the innocent third party’s *actus reus* in order to impute the crime to Michael. (For a similar ‘procuring’ case that plausibly could also have been decided under an innocent agency theory, see *R v Cogan and Leak* [1976] QB 217.) Could such an innocent agency theory be used to convict Sam of fraud on the ground that he was aware of a risk that his interference in the flow of information in the company might lead to some sort of false statement or other being unwittingly made by other employees (though he did not know which such statements would be made or where)? Likely not. There is an important difference between standard innocent agency cases and the case of Sam. While the defendant in *Michael* had the requisite *mens rea* for the offence (intent), Sam does not have the *mens rea* for fraud with respect to any particular falsehood that would be uttered by other company employees. The difficulty is that he may be aware in a general sense that the informational barriers he erects within the company will make it more likely that others will make statements that turn out to be false. But as the facts were stipulated, Sam has no awareness of any particular statement that may be false—or even of the exact report in which the falsehoods are included. This may amount to a very general form of recklessness as to the issuance of some sort of false statement by company employees in some context or other, but it is not in connection with any particularised statement or set of statements (such as the relevant report, which Sam is stipulated to be unaware of). Accordingly, while Sam seems culpable in a general sense (grounding the expectation of some criminal law response), there is a flimsy basis for coupling his very general mental state with the utterance of any particular false statement as needed to convict him of fraud on an innocent agency theory. (Indeed, doing so would be a highly worrisome precedent as it casts the net of individual criminal liability extremely widely. It would allow that any time one does something one thinks may make it more likely that others will unwittingly make false statements that benefit the company, then this opens the door to one’s own conviction for fraud. That would make criminals out of very many of us. It is for these reasons that I argue below that establishing extremely wide individual liability is not the right way to respond to cases like Version 2 (Sneaky Managers); instead, I would pursue avenues for constructing a distinct corporate *mens rea* in such cases to enable the conviction of the company only.)

52 Andrew Simester, *Fundamentals of Criminal Law* (OUP 2021) 11; AP Simester and A von Hirsch, *Crimes, Harms and Wrongs* (Hart Publishing 2011) 10–14.

53 Another practical worry is that s 46 has not been widely used by prosecutors, in part perhaps because it is so vague and expansive. Thus, expanding s 46 further may not prove so useful in practice.

54 See Ormerod and Fortson (n 8); Child (n 50); House of Commons Justice Committee, *Post-legislative Scrutiny of Part 2 (Encouraging or Assisting Crime) of the Serious Crime Act 2007* (2013–14, HC 639) <https://publications.parliament.uk/pa/cm201314/cmselect/cmjust/639/639.pdf>.

55 And even if the case does end up satisfying the elements of an expanded s 46, there is still the practical hurdle to obtaining a conviction that is created by the affirmative defence in s 50 of reasonable conduct.

56 An additional non-individualist solution, which I do not take up here, would be to consider creating a new offence that only organisations can commit, perhaps an organisational analogue of s 46. Crafting and defending the offence is not only too large an undertaking for this article, but it also has the practical drawback of requiring passing a new statute to implement.

57 This formulation is meant to navigate the issue in *R v Bainbridge* [1960] 1 QB 129, which, roughly put, suggests that for D1 to face liability for assisting D2, D1 does not need to know the particularities of D2’s crime (eg its location and timing) as long as D1 had *mens rea* as to the general type of offence involved. After all, it seems overbroad to impose liability merely for assisting ‘shady behaviour’. Under my proposed formulation, knowledge imputation to the organisation would not be supported merely by facilitating ‘bad behaviour’ understood in very vague or abstract terms; instead, some contemplation of the *criminal nature* of the harms involved is important—even though it is merely organisational liability that is at stake here, not individual liability as in *Bainbridge*.

58 See Sarch (n 10) ch 9.

59 In *Westminster CC v Croyalgrange Ltd* [1986] 1 WLR 674, 684, Lord Bridge states it is permitted ‘to base a finding of knowledge on evidence that the defendant’ was wilfully ignorant. There is debate whether this merely allows wilful ignorance to serve as evidence of knowledge or whether wilful ignorance can substitute for knowledge. However, some cases apply the latter substantive wilful ignorance rule, not merely its weaker evidential cousin. See *Ross v Moss* [1965] 2 QB 396 (DC), 406; *R v Havill* [2008] EWCA Crim 2952, [8]–[9]. In *Havill*, the Court of Appeal accepted the trial judge’s finding that defendants ‘did not know that the material they were being asked to transport consisted of’ firearms ([9]) but nonetheless approved the conviction of the offences requiring such knowledge under a wilful ignorance theory. Simester and Sullivan also are explicit that ‘wilful blindness is a doctrine of substantive rather than evidential law’. *Simester & Sullivan’s Criminal Law: Theory and Doctrine* (7th edn, Hart Publishing 2019) 166.

60 See the discussion of ‘split identification’ in nn 62–6 and accompanying text.

61 See 2023 Act, s 196.

62 Mark Dsouza dubs this approach to applying the identification doctrine ‘split identification’. Dsouza (n 33) 97.

63 ibid.

64 [1957] 1 QB 159, 172 (Denning LJ).

65
*Tesco* (n 28).

66 [1994] BCC 143, 160.

67 It contends that the identification doctrine should be extended ‘to cover situations where the conduct was done by, *or at the behest of*, a member of the senior management’. Options Paper (n 1) para 4.66 (emphasis added). Perhaps this is why the expanded identification doctrine suggested by the Law Commission was only a mental state attribution principle and did not require the manager or DMW to perform the *actus reus* herself to inculpate the company. Ibid para 4.76.

68 See also *DGFT v Pioneer Concrete* (UK) [1995] 1 AC 456, where the House of Lords found a company guilty of contempt because ‘[t]he employees … have, by their deliberate conduct, made their employers liable for disobeying the orders’ of the court (481). Here, even though management did not know that the employees entered into the agreement that violated court orders, the company was guilty of contempt (468, 475). This shows that the acts and mental state of a company’s employees can be differentiated from those of the managers or DMW of the company. Indeed, this case is more radical in fixing the company with liability even though no DMW had the *mens rea* for the offence.

69 See eg Home Office Press Release, 15 June 2023 <www.gov.uk/government/news/more-action-to-fight-fraud-bribery-and-other-economic-crime> (‘Under the proposal … senior managers will be brought within scope of who can be considered the ‘directing mind and will’ of a business’).

70 There is some interesting debate about the extent to which English courts are, or should be, willing to substantively develop the law in light of the relevant statute’s wider animating purposes or other non-textual considerations, though I cannot attempt to resolve this matter here. See eg Jonathan Green, ‘The Misunderstood History of Interpretation in England’ (draft) 1 (arguing that a pervasive approach amongst modern English courts is a kind of purposivism ‘that centers on the objective purpose of the statute’, understood as what a reasonable legislature would have held, ‘and generally ignores evidence of the subjective intentions of its authors’) <https://papers.ssrn.com/sol3/papers.cfm?abstract_id=4202843>; see also 12–13 and Part V; Richard Ekins, *The Nature of Legislative Intent* (OUP 2012) 279–84 (discussing cases where giving effect to the aims of Parliament can require corrective or equitable departures from a strict reading of the text of particular statutory provisions); Findlay Stark, ‘Judicial Development of the Criminal Law by the Supreme Court’ (2021) 41 OJLS 1, 21–4 (discussing the opportunities for and proper limits on judicial development of the criminal law as opposed to awaiting legislative action).

71 [2000] EWCA Crim 91.

72 Corporate Manslaughter and Corporate Homicide Act 2007, s 8(3).

73 [2000] EWCA Crim 91.

74 Simester (n 52).

